# The Metabolic Syndrome and ECG Detected Left Ventricular Hypertrophy – Influences from IGF-1 and IGF-Binding Protein-1

**DOI:** 10.1371/journal.pone.0108872

**Published:** 2014-12-02

**Authors:** Mats Halldin, Kerstin Brismar, Per Fahlstadius, Max Vikström, Ulf de Faire, Mai-Lis Hellénius

**Affiliations:** 1 Department of Cardiovascular Epidemiology, Institute of Environmental Medicine, Karolinska Institutet, Stockholm, Sweden; 2 Department of Molecular Medicine and Surgery, Karolinska Institutet, Rolf Luft Research Center for Diabetes and Endocrinology, Karolinska University Hospital, Stockholm, Sweden; 3 Department of Cardiology, Karolinska Institutet, Karolinska University Hospital, Stockholm, Sweden; UTHSCSH, United States of America

## Abstract

**Background and Aims:**

The metabolic syndrome (MetS) is associated with an increased risk for left ventricular hypertrophy (LVH) and cardiovascular mortality. The aim of this study was to investigate potential influences from insulin-like growth factor-1 (IGF-1) and IGF binding protein-1 (IGFBP-1) on the relationship between the MetS and LVH, also taking into account the role of physical activity (PA), use of oestrogen and gender.

**Methods and Results:**

In a population-based cross-sectional study of 60-year-old men (n = 1822) and women (n = 2049) participants underwent physical examination and laboratory tests, including electrocardiography (ECG), and completed an extensive questionnaire. Women showed higher levels of IGFBP-1 than men (37.0 vs. 28.0 µg/l, p<0.001), and women with LVH had lower levels of IGFBP-1 than women without LVH (31.0 µg/l vs. 37.0 µg/l, p<0.001). Furthermore, women with low levels of IGFBP-1 had a significantly increased risk of having LVH (crude OR≈2.5). When stratifying for PA and oestrogen, respectively, a weaker association between IGFBP-1 and LVH was demonstrated in physically active men and women, compared to inactive individuals, as well as in women using oestrogen, compared to non-users.

**Conclusion:**

In a representative sample of 60-year-old Swedish men and women, the main findings were higher levels of IGFBP-1 in women than in men; lower levels of IGFBP-1 in women with LVH, compared to women without LVH; and an increased risk of having LVH in women with low levels of IGFBP-1. The association between IGFBP-1 and LVH was diminished in physically active men and women, as well as in women using oestrogen.

## Introduction

The metabolic syndrome (MetS) is a combination of metabolic and clinical features that aggregate in individuals and increase the risk of cardiovascular disease (CVD) and mortality considerably [1]. Basically it encompasses a clustering of abdominal obesity, glucose intolerance, atherogenic dyslipidemia, and elevated blood pressure. Due to a sedentary lifestyle, an increasing prevalence has been observed worldwide; approximately one fourth of the adult population in Europe, United States and Latin America meet its diagnostic criteria [2]. In East Asian countries, including China, Japan and Korea, the MetS is considered “an emerging epidemic”, with prevalences ranging from 8–13% in men and from 2–18% in women [3].

The increased CVD risk may be related to the development of more pronounced subclinical alterations in cardiac and vascular structures and function in individuals with MetS. Left ventricular hypertrophy (LVH), for example, is more prevalent among individuals with MetS [4].

LVH is a potent independent predictor of cardiovascular morbidity and mortality, and blood pressure overload is the pivotal risk factor for its development – but many other factors, including gender, age, body mass index, diabetes mellitus and level of physical activity are potential determinants of LVH.

Furthermore, insulin-like growth factor-1 (IGF-1), an endocrine hormone mainly stimulated by growth hormone, and structurally and functionally related to insulin, is an important mediator of cell growth, and may therefore also induce cardiac hypertrophy. Although it is widely accepted that the heart is an end organ of growth hormone/IGF-1 action [5], contradictive studies have been published [6].

Less than 1% of the total serum IGF-1is freely circulating; the remainder is bound to six high-affinity insulin-like growth factor binding proteins (IGFBPs), which play an important role in regulating IGF-1 activity, having both stimulatory and inhibitory effects. IGFBPs may also have IGF-independent effects [7].

In a previous study on the relationship between MetS and LVH we observed that among the various components in MetS, hypertension was most strongly and independently related to LVH in both men and women. In women, however, also abdominal obesity, high glucose levels and hyperinsulinaemia were related to LVH, suggesting a gender differences in the mechanisms behind development of LVH [8].

Hence, the aim of this study was to investigate potential influences from IGF-1 and IGFBP-1 on the relationship between the MetS and LVH in a population based representative cohort of 60-year old men and women. Furthermore, the role of physical activity (PA), use of oestrogen and gender were addressed.

## Methods

### Study Population

From August 1997 to March 1999, every third person (5460 individuals in total, 2681 men and 2779 women) living in Stockholm County, Sweden, born between 1 July 1937 and 31 June 1938, was invited to participate in a health screening survey. All participants underwent a physical examination collecting weight, height, waist circumference and sagittal abdominal diameter (SAD). Systolic and diastolic blood pressures were measured twice in the sitting position, after five minutes of rest in supine position, and the mean of the measurements was calculated. An automatic device was used (HEM 711, Omron Healthcare, Bannockburn, IL, USA). In participants with an upper arm circumference above 32 cm, a wider cuff was used.

Fasting blood samples were taken and a comprehensive questionnaire was completed. The study was approved by the ethical committee at the Karolinska Institutet. All of the participants gave their written consent.

### Blood analyses

Venous blood samples were drawn from an antecubital vein after overnight fasting. All blood samples were analysed online (continuously). Triglycerides and cholesterol in serum were analysed using enzymatic methods (Bayer Diagnostics, Tarrytown, NY, USA) [9], [10]. HDL in serum was measured after isolation of LDL and VLDL (Boehringer Mannheim Gmbh, Germany) and LDL was estimated using Friedewald's method [11]. Serum glucose was measured with an enzymatic colorimetric test (Bayer Diagnostics, Tarrytown, NY, USA), and serum insulin levels were determined using the ELISA technique (Boeringer Mannheim Gmbh, Diagnostica, Germany). Total IGF-1 was determined in serum by an in-house radioimmunoassay (RIA) after separation of IGFs from IGFBPs by acid ethanol extraction and cryoprecipitation. To minimise interference of remaining IGFBPs, des (1–3) IGF-1 was used as radioligand [12]. The intra- and interassay CV were 4% and 11%, respectively. Serum levels of IGF-1 are age dependent, decreasing which age, thus IGF-1 values are also expressed as standard deviation (SD) scores, calculated from the regression of the values of 247 and 448 healthy adult subjects, respectively [13], [14]. IGFBP-1 concentrations in serum were determined by an in-house RIA according to the method by Póvoa et al. [15]. The sensitivity of the RIA was 3 µg/l and the intra- and interassays CV were 3% and 10%, respectively.

### Physical activity (PA)

PA in leisure-time during the past year was asked for in a questionnaire, and the participants classified themselves into one of four groups:

(1) low PA, a sedentary lifestyle with less than 2 hours of light PA/week (i.e. walking, cycling);

(2) light PA (generally without sweating) at least 2 hours/week (i.e. walking, gardening, fishing);

(3) moderate PA, regular activity 1–2 times/week, at least 30 minutes each time (i.e. jogging, swimming, tennis); and

(4) high PA, intensive regular activity more than 2 times/week, at least 30 minutes each time (i.e. running, swimming, aerobics or other strain exercise).

### Left ventricular hypertrophy

LVH was defined by standard 12-lead resting electrocardiograms (ECG) using two established criteria for LVH, the Minnesota Code and the Cornell voltage-duration product. ECG measurements were made with a ruler (“nomogram”) on the resting ECG tracings.

The Minnesota Code for LVH was based on class 3∶1, continuing to 3∶3 if 3∶1 was not fulfilled [16]. Either the Minnesota Code or Cornell voltage-duration had to be positive for LVH to qualify for LVH in this work.

Ten percent of the samples (n = 400, randomly selected) were validated by Professor Sverker Jern's research group in Gothenburg, Sweden. The validation showed a correspondence of 83% between the two evaluations (Stockholm versus Gothenburg).

### Metabolic syndrome

MetS was classified using the criteria proposed by the American Heart Association/National Heart, Lung, and Blood Institute [17], [18], where three or more of the following criteria were applied: (1) fasting plasma glucose concentration of 5.6 mmol/l (100 mg/dl) or greater or on drug treatment for elevated glucose; (2) a triglyceride concentration of 1.7 mmol/l (150 mg/dl) or greater or on drug treatment for elevated triglycerides; (3) a HDL concentration less than 1.0 mmol/l (40 mg/dl) in men and less than 1.3 mmol/l (50 mg/dl) in women or on drug treatment for reduced HDL; (4) a systolic blood pressure of 130 mm Hg or greater or a diastolic blood pressure of 85 mm Hg or greater or on antihypertensive drug treatment in a patient with a history of hypertension; and (5) a waist circumference of 102 cm or greater in men and 88 cm or greater in women.

### Statistics

For between-group analysis, t-tests or, when data had a skewed distribution, the Mann-Whitney *U* test was used. Crude and adjusted (not presented) OR:s were calculated for the MetS and its different components, as well as for insulin, IGF-1, IGFBP-1, oestrogen and PA. In the logistic regression models, PA group 3 and 4 (above) were referred to as “high” PA. The “low” PA-group (group 1 and 2) was set as a reference. All statistical analyses were performed using SAS statistical software system version 9.2.

## Results

Altogether, 4228 individuals participated (2036 men and 2192 women) in the survey, which corresponds to a response rate of 77%. After exclusions of 357 individuals with reported myocardial infarction (n = 110), angina pectoris (n = 148), heart failure (n = 53), intermittent claudication (n = 73), and/or stroke (n = 60), 1822 men and 2049 women remained for the present investigation.

### Characteristics of the study population

The characteristics of the survey population are described in detail elsewhere [8]. Anthropometric characteristics and biochemical measurements of men and women with or without LVH are presented in Table 1. In general, women with LVH revealed a more metabolically deranged status than men, while systolic as well as diastolic blood pressures were generally higher in men.

**Table 1:** Study population: anthropometric characteristics and biochemical measurements in relation to gender and occurrence of left ventricular hypertrophy (LVH).

	Men (n 1822)			Women (n 2049)		
	No LVH (n 1649)	LVH (n 173)	*p*-value	No LVH (n 1936)	LVH (n 113)	*p*-value
**Waist (cm)**	97.2 (±10.3)	98.4 (±10.8)	0.178	85.9 (±11.6)	91.0 (±13.2)	<0.001
**SAD (cm)**	21.3 (±2.7)	21.7 (±3.0)	0.088	19.7 (±2.7)	20.6 (±3.1)	0.002
**SBP (mmHg)**	141 (±19)	156 (±24)	<0.001	133 (±21)	151 (±24)	<0.001
**DBP (mmHg)**	87 (±10)	93 (±12)	<0.001	81 (±10)	89 (±9)	<0.001
**TG (mmol/l)** *	1.2 (0.8; 1,7)	1.1 (0.9; 1.8)	1.000	1.1 (0.8; 1.5)	1.1 (0.8; 1.6)	1.103
**TChol (mmol/l)**	5.8 (±1.0)	5.9 (±1.0)	0.266	6.1 (±1.0)	6.4 (±1.7)	0.008
**HDL (mmol/l)**	1.3 (±0.3)	1.3 (±0.4)	0.460	1.6 (±0.4)	1.6 (±0.4)	0.529
**LDL (mmol/l)**	3.8 (±0.9)	3.9 (±0.9)	0.496	5.1 (4.7; 5.5)	5.3 (4.9; 5.8)	<0.001
**Glucose (mmol/l)** *	5.4 (5.0; 5.9)	5.4 (5.0; 6.0)	0.488	5.1 (4.7; 5.5)	5.3 (4.9; 5.8)	<0.001
**Insulin (µU/ml)** *	8.9 (6.7; 12.8)	9.8 (7.3; 14.4)	0.042	8.4 (6.2; 11.2)	10.6 (7.0; 14.1)	<0.001
**IGFBP-1 (µg/l)** *	28.0 (20; 40)	26.0 (18; 41)	0.376	37.0 (28; 49)	31.0 (22; 46)	<0.001
**IGF-1 (µg/l)**	161.5 (±49.7)	164.5 (±51.0)	0.454	151.0 (±46.3)	152.4 (±46.3)	0.756

Footnote: Values are means (±SD). SAD, sagittal abdominal diameter; SBP, systolic blood pressure; DBP, diastolic blood pressure; TG, triglycerides; TChol, total cholesterol; HDL, high-density lipoprotein cholesterol; LDL, low-density lipoprotein cholesterol; IGFBP-1, insulin-like growth factor binding protein-1; IGF-1, insulin-like growth factor-1.

*Skewed distribution (median, percentiles).

The levels of IGFBP-1 demonstrated a significant (*p*<0.001) gender difference, with generally higher levels of IGFBP-1 in women (37.0 vs. 28.0 µg/l, on average). When discriminating between individuals with and without LVH, a significant difference in levels of IGFBP-1 was observed in women (31.0 vs. 37.0 µg/l, *p*<0.001), but not in men. Concerning the levels of IGF-1, a significantly (*p*<0.001) higher level was seen in men compared to women (161.8 vs. 151.1 µg/l, on average). No significant difference was observed between individuals with and without LVH, neither in men nor in women.

### The risk of having LVH

The risk for LVH in relation to the MetS and its different components, as well as insulin, level of leisure-time physical activity, use of oestrogen, and percentile levels of IGFBP-1 and IGF-1 are presented in Table 2.

**Table 2:** The risk of having left ventricular hypertrophy (crude odds ratio) for the metabolic syndrome and its different components and insulin as well as physical activity, oestrogen and percentile levels of insulin-like growth factor binding protein-1 and insulin-like growth factor-1.

	Men (n 1822)			Women (n 2049)		
	N	Crude OR	95% CI	N	Crude OR	95% CI
**MetS**	492	1.63	**1.17–2.26**	416	2.37	**1.59–3.54**
**Waist (>102/88 cm)**	495	1.44	**1.03-2.01**	784	2.21	**1.50-3.24**
**BP (>130/85 mmHg)**	1348	3.52	**2.11-5.87**	1128	4.61	**2.77–7.70**
**TG (>1.7 mmol/l)**	476	1.06	0.75**–**1.51	368	1.55	0.99**–**2.41
**HDL (<1.03/1.3 mmol/l)**	330	1.21	0.82**–**1.79	380	1.00	0.62**–**1.63
**Glucose (≥5.6 mmol/l)**	615	1.30	0.95**–**1.80	381	2.79	**1.87**–**4.15**
**Insulin (>11.1/10.2 µU/ml)**	617	1.37	0.99**–**1.89	689	2.30	**1.57**–**3.38**
**PA (high)**	611	1.19	0.85**–**1.66	547	1.04	0.67**–**1.61
**Oestrogen**	-	-	-	549	0.47	**0.28**–**0.79**
**IGFBP-1(10)**	221	1.04	0.57**–**1.90	248	2.56	**1.23**–**5.83**
**IGFBP-1(20)**	180	0.70	0.35**–**1.38	195	2.43	**1.03**–**5.72**
**IGFBP-1(30)**	168	0.80	0.41**–**1.58	204	1.49	0.60**–**3.73
**IGFBP-1(40)**	165	0.88	0.45**–**1.71	196	1.42	0.56**–**3.61
**IGFBP-1(50)**	193	0.60	0.30**–**1.21	219	0.67	0.23**–**1.97
**IGFBP-1(60)**	209	0.55	0.28**–**1.11	179	1.12	0.41**–**3.04
**IGFBP-1(70)**	154	0.55	0.26**–**1.18	205	0.97	0.36**–**2.64
**IGFBP-1(80)**	201	0.66	0.34**–**1.30	211	1.44	0.58**–**3.60
**IGFBP-1(90)**	160	0.74	0.37**–**1.49	199	0.77	0.26**–**2.25
**Ref (100)**	**171**	**1**		**193**	**1**	
**IGF-1(10)**	189	0.91	0.45**–**1.85	201	1.09	0.51**–**2.33
**IGF-1(20)**	179	0.53	0.23**–**1.20	220	0.75	0.32**–**1.76
**IGF-1(30)**	197	0.97	0.50**–**1.90	207	0.30	**0.10**–**0.92**
**IGF-1(40)**	190	1.24	0.64**–**2.41	210	1.08	0.50**–**2.36
**IGF-1(50)**	175	0.91	0.45**–**1.85	212	0.90	0.40**–**2.03
**IGF-1(60)**	173	0.94	0.47**–**1.87	198	0.75	0.32**–**1.76
**IGF-1(70)**	181	1.01	0.50**–**2.04	193	1.15	0.53**–**2.49
**IGF-1(80)**	181	1.06	0.54**–**2.10	214	0.47	0.18**–**1.26
**IGF-1(90)**	179	0.96	0.48**–**1.91	192	0.97	0.44**–**2.14
**Ref (100)**	**182**	**1**		**202**	**1**	

Footnote: MetS, metabolic syndrome; BP, blood pressure; TG, triglycerides; HDL, high-density lipoprotein cholesterol; PA, physical activity; IGFBP-1, insulin-like growth factor binding protein-1; IGF-1, insulin-like growth factor-1.

Hypertension was most strongly and independently related to LVH in both men and women. In women, also abdominal obesity, high glucose levels and hyperinsulinaemia were independently related to LVH. Oestrogen (in women) was significantly negatively associated with LVH, with a crude OR of 0.47 (0.28–0.79).

In women, low percentile levels of IGFBP-1 were significantly associated with LVH. In men, no significant association between IGFBP-1 and LVH was observed. Although not significant, there were generally higher OR:s for the risk of LVH in women throughout all percentile levels of IGFBP-1.

The analysis of IGF-1, also based on percentile levels, did not yield any significant association to LVH (except for a negative association in the 30^th^ percentile in women), neither in men nor in women.

**Figure 1:**
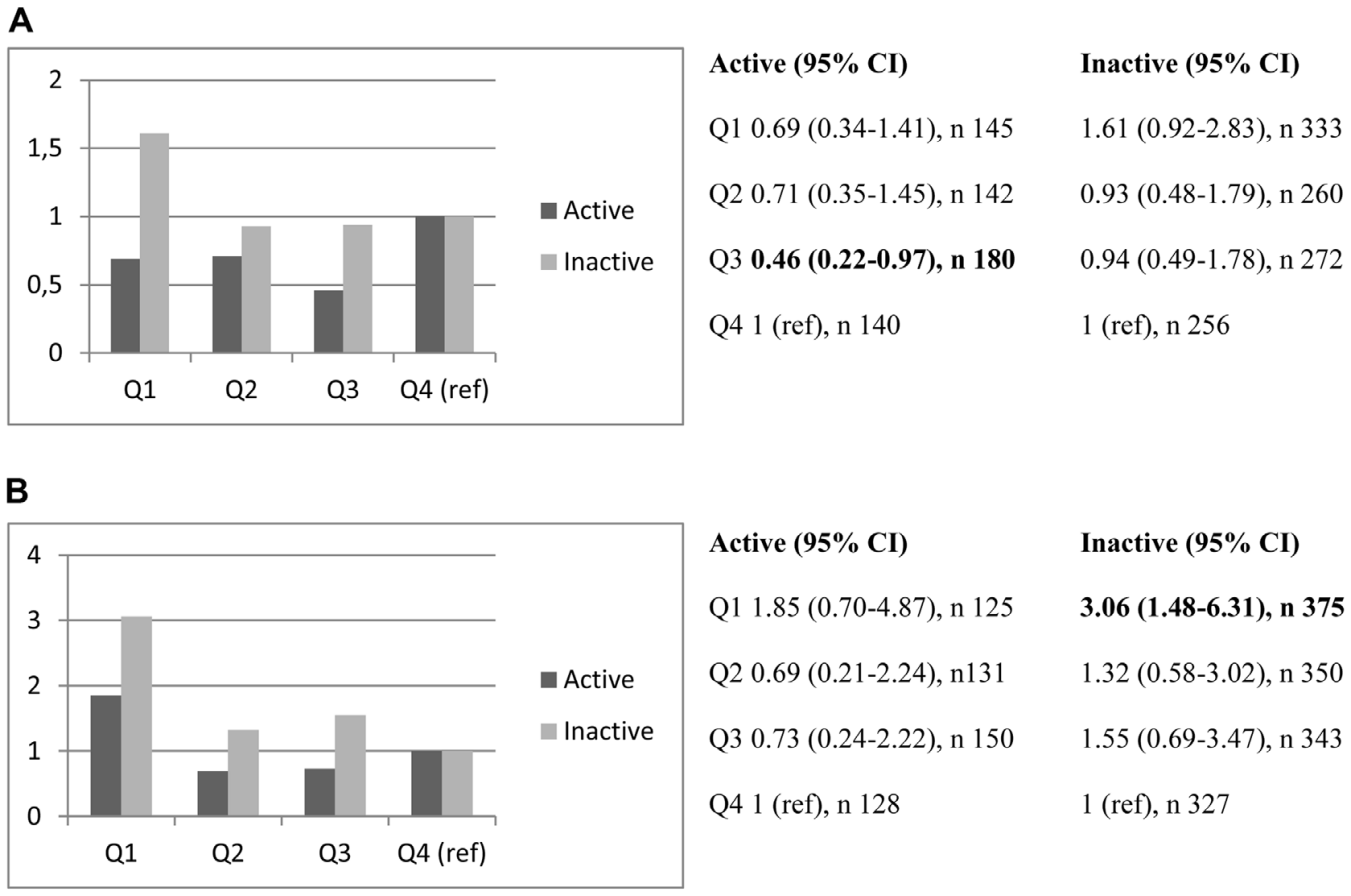
a. Risk of having LVH (crude OR) in different quartiles of IGFBP-1 in physically active and inactive men, respectively. **b**. Risk of having LVH (crude OR) in different quartiles of IGFBP-1 in physically active and inactive women, respectively.

When stratifying the risk of LVH for physical activity, presented as quartiles of IGFBP-1, there was a relatively weaker association (crude OR) to LVH in active men and women – especially in the lowest quartile of IGFBP-1 in both genders (Figure 1a and 1b).

Furthermore, the risk of LVH was significantly higher in physically inactive women in the lowest quartile of IGFBP-1, compared to the highest quartile (set as reference).

**Figure 2:**
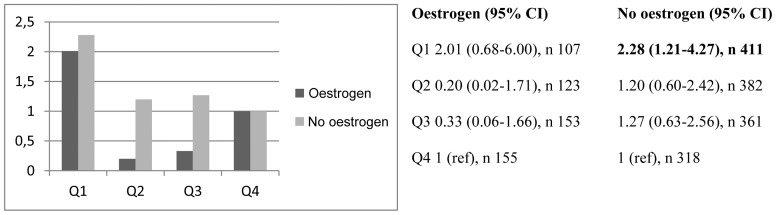
Risk of having LVH (crude OR) in different quartiles of IGFBP-1 in women with and without reported use of oestrogen, respectively.

When stratifying the risk of LVH for oestrogen, we found a generally lower risk among users (Figure 2). The crude OR in the lowest quartile of non-users was significantly associated with LVH (OR 2.28, CI 1.21–4.27).

## Discussion

### Main findings

In a representative sample of 60-year-old Swedish men and women, the salient findings were significantly higher levels of IGFBP-1 in women than in men; significantly lower levels of IGFBP-1 in women with LVH, compared to women without LVH; significantly increased risk of having LVH among women with the lowest levels of IGFBP-1; and a significantly negative association between use of oestrogen in women and occurrence of LVH in the crude model.

When stratifying for physical activity, a generally weaker association between IGFBP-1 and LVH in physically active participants was observed, along with a significantly higher risk of LVH in the lowest quartile of IGFBP-1 in women.

In a stratified model of oestrogen-use, the risk of LVH was lower throughout all quartiles of IGFBP-1 (except for the reference quartile, Q4) among users – and significantly *higher* in the lowest quartile of non-users (compared to Q4).

### Comparisons with previous research

Generally higher levels of IGFBP-1 in women have been described in previous studies [19], [20]. The levels were explained by insulin, body mass index (BMI) and waist circumference but also by oestrogens. IGFBP-1 may act as a measure of hepatic insulin resistance as well as whole body insulin resistance [21], [22]. Furthermore, a series of epidemiological studies during the past two decades has linked low circulating IGFBP-1 concentrations with insulin resistance, type 2 diabetes, and CVD [23].

Concerning associations between oestrogen and LVH, there are somewhat inconsistent findings. However, there seems to be beneficial effects from oestrogen (i.e. hormone replacement therapy) on the development of LVH (and/or left ventricular mass) in a majority of the published studies [24], [25], [26]. One study concludes that the differences in the presumptive protective benefit of menopausal hormone therapy between never-users and users partly could be explained by lifestyle or health-related factors, related to the use of hormones [27].

### Potential mechanisms

LVH is recognized as an independent risk factor for CVD, and represents a powerful independent predictor of cardiovascular morbidity and mortality in the general population. LVH is only partly a result from hypertension (although high blood pressure seems to be the strongest predictor of LVH), and has been shown to be of a more complex and multifactorial origin. Insulin, for example, has anabolic effects on smooth muscle cells, and may directly induce LVH by binding of insulin to the IGF-1 receptors expressed in the myocardium. Elevated plasma insulin is associated with LVH, and may play an important role in the development and progression of LVH, especially in females [28], [29], [30]. IGFBP-1 is regulated at transcriptional level by insulin and low fasting levels are a marker of hyperinsulinemia. Furthermore, IGFBP-1 is the most important dynamic regulator of free IGF-1 activity [31].

Low IGFBP-1 per se may play an important role in the development of LVH, since it may enhance the effect of IGF-1, but also by its own direct effect on proliferation and migration through binding to the alpha 5 beta 1 integrin receptor [32].

The observed beneficial effects on LVH in oestrogen-users have several possible reasons, besides the direct hormonal effects (i.e smooth muscle relaxant as well as antiproliferative effects). For example, key enzymes in glucose and fatty acid metabolism are regulated by oestrogens and, consequently, obesity-associated conditions such as diabetes are particularly associated with development of LVH. Moreover, menopausal hormone therapy users are likely to be better educated, more physically active, and have a lower BMI and blood pressure than untreated women because of a more health-conscious behavior.

### Strengths and limitations

The design of this study is based on a large and representative cohort, from every third man and women in an urban population in Stockholm County in 1997–99 with a participation rate of approximately 77%. The cohort is thoroughly characterized throughout a well-defined questionnaire and physical examinations. As the cohort includes both men and women it allows a focus on gender perspectives. Although information from self-reports has been questioned, questionnaires to assess habitual PA may give valid and reliable data [33], [34].

The cross-sectional design cannot prove causality, but only describe associations as they exist at a particular point in time, and hence generate hypotheses. As all participants in our study are 60 years old, the interpretation of the results should be restricted to men and women around this age only.

Concerning the reported use of oestrogen, it has to be taken into consideration that the substitution includes oral oestrogen replacement therapy (ERT) as well as locally/transdermally administered ERT. As the serum levels of oestrogen after local treatment likely are relatively low, the effects of reported oestrogen-use may be underestimated. On the other hand, oestrogen-users may be generally healthier compared to non-users, which might have caused an overestimation of the potentially protective effects of the hormone.

Furthermore, LVH determined by ECG might be a limitation, compared to echocardiographic examinations. However, the ECG has been used in population studies for over 50 years, becoming one of the most important non-invasive and reproductible imaging methods in the evaluation of cardiac morphology and dynamics [35], and many studies have confirmed the high specificity of ECG criteria for the diagnosis of LVH [36]. Moreover, obesity may limit sensitivity of ECG voltage criteria for LVH because of the attenuating effects of increased body mass on precordial voltages. However, Cornell product criteria for ECG LVH appear to provide a relatively accurate measure of LVH in obese and overweight individuals [37]. In this study, 10% of the ECG measurements were validated by experienced expertise showing a satisfyingly correspondence (83%).

### Clinical aspects

The current study indicates that the pathogenesis of LVH is complex and depends on both hemodynamic and metabolic conditions as well as gender. Therefore it is important to take into account not only MetS components, like blood pressure and glucose/insulin levels, when analysing potential CVD risk factors – but also parameters like IGFBP-1, oestrogen and physical activity.

### Conclusions

In a representative sample of 60-year-old Swedish men and women, there was a clear gender difference not only in the levels of IGFBP-1, but also when relating IGFBP-1 to the risk of having LVH. The gender difference noted may partly be due to the use of oestrogen, but metabolic factors like hyperglycemia and hyperinsulinaemia are most likely also in operation.

Finally, leisure-time physical activity seemed to diminish the strong association between (especially lower) levels of IGFBP-1 and LVH in both men and women. The use of oestrogen and levels of physical activity, together with levels of IGFBP-1, are parameters to consider in the clinical setting, to be able to prevent deleterious consequences of LVH on CVD morbidity and mortality. In this study levels of total IGF-1 did not yield any firm relationships to LVH.

Further elucidation of the biological processes linking the IGF-1/IGFBP-1 systems to risk of LVH will be important in facilitating the identification of people at risk for CVD.
